# Tribute to Our Reviewers – 2022

**DOI:** 10.1002/deo2.224

**Published:** 2023-03-21

The Editorial Board of *DEN Open* takes this opportunity to acknowledge with particular gratitude the following reviewers who have provided their valuable time and expertise in the evaluation of manuscripts submitted to *DEN Open* during the period of January 1, 2022, to December 31, 2022.

Takao Itoi

Editor‐in‐Chief

*DEN Open*

Nobutsugu Abe

Yoichi Akazawa

Ikuo Aoyama

Kyohei Ariake

Reiko Ashida

Tesshin Ban

Shannon Chan

Hideyuki Chiba

Han‐Mo Chiu

Hourin Cho

Akira Dobashi

Osamu Dohi

Shinpei Doi

Luca Elli

Hiroyuki Endo

Mitsuru Esaki

Nao Fujimori

Toshio Fujisawa

Kazushi Fukagawa

Mitsuharu Fukasawa

Norimasa Fukata

Nobuhiko Fukuba

Shuichi Fukuda

Shusei Fukunaga

Masakatsu Fukuzawa

Naotake Funamizu

Yoshihiro Furuichi

Atsushi Goto

Natalie Halvorsen

Noboru Hanaoka

Kazuo Hara

Hideaki Harada

Ryo Harada

Shinichi Hashimoto

Takemasa Hayashi

Yukie Hayashi

Takuma Higurashi

Susumu Hijioka

Takuto Hikichi

Kingo Hirasawa

Daizen Hirata

Takashi Hirose

Takashi Hisabe

Shinichiro Hori

Masayasu Horibe

Yusuke Horiuchi

Kinichi Hotta

Ryoji Ichijima

Satoshi Ikarashi

Yuichiro Ikebuchi

Koji Ikeda

Hisatomo Ikehara

Kenji Ikezawa

Atsushi Imagawa

Kenichiro Imai

Yutaka Inada

Tadahisa Inoue

Yusuke Ishida

Noriyoshi Ishikawa

Hirotoshi Ishiwatari

Misaki Ishiyama

Hirotaka Ito

Ken Ito

Nobuhito Ito

Masahiro Itonaga

Takuji Iwashita

Taro Iwatsubo

Tomohiro Kadota

Yasuo Kakugawa

Hideki Kamada

Tomoari Kamada

Ken Kamata

Takashi Kanesaka

Shuji Kanmura

Atsushi Kanno

Takeshi Kanno

Yoshihide Kanno

Hiromitsu Kanzaki

Motohiko Kato

Koichiro Kawaguchi

Kousaku Kawashima

Daisuke Kikuchi

Toshifumi Kin

Tetsu Kinjo

Satoshi Kinoshita

Katsuya Kitamura

Hideki Kobara

Hirofumi Kogure

Tomoyuki Koike

Yoriaki Komeda

Yoshiyasu Kono

Shinsuke Koshita

Yuto Kubo

Masaya Kubota

Takahiro Kudo

Hiroki Kurumi

Ryusaku Kusunoki

Masaki Kuwatani

Yuki Maeda

Mai Makiguchi

Daisuke Maruoka

Akinori Maruta

Tatsuhiro Masaoka

Saburo Matsubara

Carolina Matsubayashi

Shigenaga Matsui

Kazuyuki Matsumoto

Tomoaki Matsumura

Noriko Matsuura

Kosuke Minaga

Kenichi Mochizuki

Jong Ho Moon

Rintaro Moroi

Shuntaro Mukai

Takashi Murakami

Tatsuro Murano

Yasuaki Nagami

Itaru Naitoh

Kazunari Nakahara

Yousuke Nakai

So Nakaji

Keiichiro Nakajo

Jun Nakamura

Masanao Nakamura

Yoshiko Nakayama

Masato Narita

Hiroko Nebiki

Tsutomu Nishida

Noriko Nishiyama

Toshihiro Nishizawa

Satoru Nonaka

Haruei Ogino

Masayasu Ohmori

Masayuki Ohta

Kazuo Ohtsuka

Naoki Okano

Taishi Okumura

Nozomi Okuno

Toru Okuzono

Teppei Omori

Shunsuke Omoto

Satoshi Osawa

Shozo Osera

Masahiro Saito

Tomotaka Saito

Yoshiki Sakaguchi

Akira Sakamaki

Yoji Sanomura

Fumisato Sasaki

Yu Sasaki

Hiroki Sato

Hiroaki Sawai

Ryo Seishima

Masau Sekiguchi

Yuto Shimamura

Hideyuki Shiomi

Yohei Shirakami

Yoshii Shunsuke

Antonio Sterpetti

Mitsushige Sugimoto

Tetsuya Sumiyoshi

Haruhisa Suzuki

Nobumi Tagaya

Masahiro Tajika

Kaoru Takabayashi

Tadayuki Takagi

Hiroyuki Takamaru

Yuki Takashina

Yusaku Takatori

Yohei Takeda

Mamoru Takenaka

Kyosuke Tanaka

Yuki Tanisaka

takeshi tomoda

Ryosuke Tonozuka

Nobuyuki Toshikuni

Yosuke Toya

Takayoshi Tsuchiya

Yosuke Tsuji

Shunsuke Tsukamoto

Jason A. Tye‐Din

Junji Umeno

Yuji Urabe

Kenji Watanabe

Yukinori Yamagata

Daisuke Yamaguchi

Katsumi Yamamoto

Shunsuke Yamamoto

Yorimasa Yamamoto

Yoshinobu Yamamoto

Kentaro Yamao

Kei Yane

Shigetaka Yoshinaga

Toshiyuki Yoshio

